# Imaging of the Intestinal Microcirculation during Acute and Chronic Inflammation

**DOI:** 10.3390/biology9120418

**Published:** 2020-11-26

**Authors:** Kayle Dickson, Hajer Malitan, Christian Lehmann

**Affiliations:** 1 Department of Microbiology and Immunology, Dalhousie University, Halifax, NS B3H 4R2, Canada; kayle.dickson@dal.ca; 2 Department of Anesthesia, Pain and Perioperative Management, Dalhousie University, Halifax, NS B3H 4R2, Canada; hajer.malitan@dal.ca; 3 Department of Physiology and Biophysics, Dalhousie University, Halifax, NS B3H 4R2, Canada; 4 Department of Pharmacology, Dalhousie University, Halifax, NS B3H 4R2, Canada

**Keywords:** inflammation, microcirculation, intravital microscopy

## Abstract

**Simple Summary:**

Microcirculation refers to the smallest blood vessels within the body. During inflammation, changes can occur within these vessels which can further disease processes. Blood vessels in the gut are particularly vulnerable. Videomicroscopy devices are important for examining these changes. In animal experiments, intravital microscopy is the gold standard for evaluation. This technique allows for the visualization of these vessels within living animals. The changes that occur vary depending on the length of time the inflammation has been occurring for. Examples of these changes include changes in blood flow, vessel density and immune cell activation. This review discusses these changes in the context of various inflammatory conditions including infections of the intestine and pancreas, and non-infectious conditions of the bowel.

**Abstract:**

Because of its unique microvascular anatomy, the intestine is particularly vulnerable to microcirculatory disturbances. During inflammation, pathological changes in blood flow, vessel integrity and capillary density result in impaired tissue oxygenation. In severe cases, these changes can progress to multiorgan failure and possibly death. Microcirculation may be evaluated in superficial tissues in patients using video microscopy devices, but these techniques do not allow the assessment of intestinal microcirculation. The gold standard for the experimental evaluation of intestinal microcirculation is intravital microscopy, a technique that allows for the in vivo examination of many pathophysiological processes including leukocyte-endothelial interactions and capillary blood flow. This review provides an overview of changes in the intestinal microcirculation in various acute and chronic inflammatory conditions. Acute conditions discussed include local infections, severe acute pancreatitis, necrotizing enterocolitis and sepsis. Inflammatory bowel disease and irritable bowel syndrome are included as examples of chronic conditions of the intestine.

## 1. Introduction

Microcirculation refers to the circulation of blood through microvessels with a diameter of less than 100 μm, which include arterioles, capillaries and venules. Larger vessels from the macrocirculation, including arteries and veins, supply blood to the microcirculation. The microcirculation has many roles within the body, as reviewed in detail by Guven et al. [1]. The main role of the microcirculation is to distribute oxygen and nutrients within tissues. Additionally, microcirculation is responsible for the mediation of immune function and hemostasis. Microcirculatory blood flow is regulated primarily by vascular smooth muscle and responds to signals from the nervous system, hormones, and metabolic stimuli.

**Figure 1:**
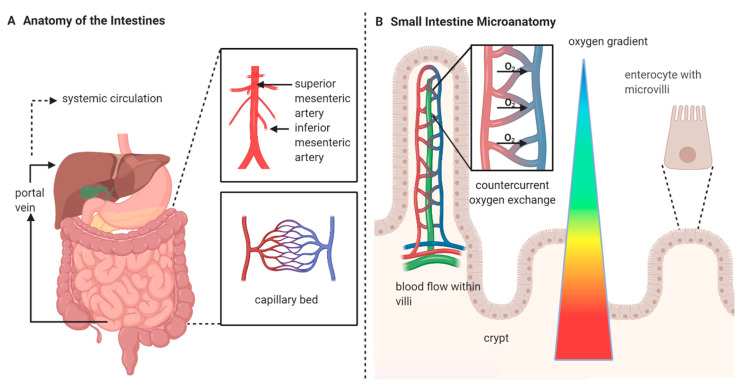
Anatomy of the gastrointestinal (GI) tract. (**A**) The macrocirculation and general anatomy of the GI tract are shown. Major arteries and the portal vein are indicated, and the generic structure of a capillary bed within intestine is shown. (**B**) The microanatomy of the small intestine is shown. The small intestine features protruding villi and crypts which are lined with enterocytes. Microvilli are located on the tips of enterocytes. Within the villi, arterial and venous circulation run parallel to each other, facilitating the diffusion of oxygen into the vein. This countercurrent oxygen exchange within the villi results in a descending partial pressure of oxygen from the tip to the base of the villi.

The gastrointestinal (GI) tract is supplied by three major arteries, with the superior and inferior mesenteric arteries supplying the small and large intestine. A large number of anastomoses are present to facilitate continuous flow through the intestine. Intramural blood flow is not uniform, with the mucosal layer receiving 70–80% of the blood supply depending on food intake [2]. Not all capillaries are continuously supplied, as mechanisms exist to recruit additional capillaries are needed for oxygenation, nutrient delivery and waste removal. Venous return drains into the portal vein for recirculation to the heart. Several factors contribute to the sensitive and unique nature of microcirculatory blood flow in the GI tract. The intestine has unique microanatomy, where the artery and vein within the villi run parallel to each other, which results in low oxygenation in the most luminal areas of the intestine, even under optimal conditions (Figure 1). Oxygen homeostasis in the healthy intestine, which features the countercurrent exchange of oxygen, produces a state of physiologic hypoxia which leaves the intestine vulnerable to microcirculatory disturbances [3]. This effect is less pronounced within the large intestine, which lacks villi. During physiological stress, the release of catecholamines such as norepinephrine results in vasoconstriction and redistribution of blood flow from the both the small and large intestines to vital organs. In general, no major differences are expected between the small and large intestines with respect to microcirculatory function, except those due to differences in the anatomy (e.g., the absence of villi in the large intestine).

**Figure 2:**
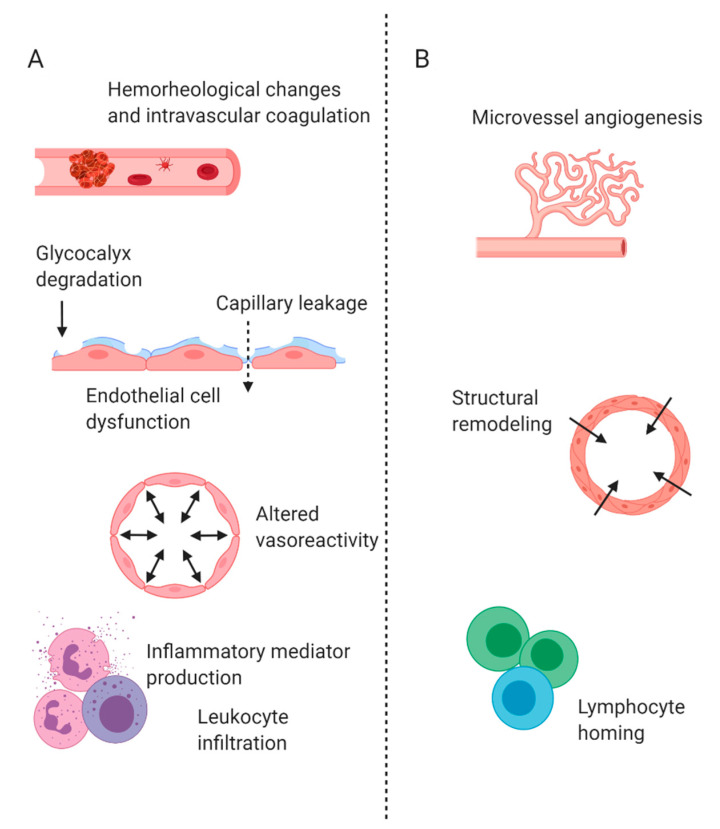
Microcirculatory changes during (**A**) acute and (**B**) chronic inflammation of the intestine. (**A**) During acute inflammation, various microcirculatory changes occur. Hemorheological changes and intravascular coagulation impact blood flow. Degradation of the glycocalyx and capillary leakage contribute to endothelial cell dysfunction. Microvessels may also experience altered vasoreactivity. In addition, leukocytes become highly activated and may infiltrate into tissues and produce high levels of inflammatory mediators. (**B**) During chronic inflammation, additional microcirculatory changes may occur. Microvessel angiogenesis and structural remodeling impact vessel structures. Lymphocyte homing occurs as the adaptive immune system responds.

In a state of homeostasis, macro and microcirculation are coupled to each other (i.e., hemodynamic coherence). Inflammation, either local or systemic, may result in microcirculatory disturbances and ultimately in the uncoupling of these circulatory systems. Because of the uncoupling of macro and microcirculation, therapies aimed at restoring microcirculatory parameters may not be effective at a microcirculatory level [4]. Acute inflammation (Figure 2A) results in functional and structural changes including endothelial cell dysfunction, glycocalyx degradation, hemorheological changes and altered vasoreactivity, which impact microcirculatory function [1,5]. Further changes occur during chronic inflammation (Figure 2B), including structural changes to the microvessels such as angiogenesis and vascular remodeling [6]. All these alterations may impact microcirculatory flow. As reviewed by Ince, four main types of microcirculatory alterations are possible: heterogenous flow, reduced capillary density, flow reduction and tissue edema from capillary leakage [5]. These changes all reduce the functional capillary density (FCD) within organs and compromise oxygen delivery. Microcirculatory alterations have been noted in various acute and chronic inflammatory conditions affecting the gut, including local infections, sepsis and chronic inflammatory disorders of the gastrointestinal (GI) tract. 

Two key parameters are established for assessing the microcirculation. Capillary density and blood flow can be assessed directly using a variety of microscopy techniques. Assessment of the microcirculation in patients is limited to areas with superficial vasculature such as sublingual mucosal tissue. Accurate assessment of the microcirculation of internal organs, such as the intestine, remains challenging. Changes in the sublingual microcirculation are known to parallel those of the intestine, but this data may not always be representative of the intestinal microcirculation [7]. Rectal microcirculation has also been used as a surrogate marker for microcirculatory changes in the GI tract [8]. Various imaging techniques are available for evaluating microcirculation, including videomicroscopy, laser Doppler flowmetry and laser speckle contrast imaging. Handheld microscopes, utilizing sidestream dark-field or incident dark-field imaging techniques, are frequently used to assess sublingual microcirculation. These techniques are reviewed in detail elsewhere [9,10]. It is important to note that these techniques are not currently approved for clinical use. The development and clinical implementation of such techniques is a current priority in critical care [11]. One barrier to implementation is a lack of reliable automated systems, as current technologies require manual video analysis. A recent study comparing the automated CCTools^®^ software to the semiautomated AVA3^®^ technique noted that while the parameters are statistically related, further development is needed before the implementation of the fully automated software [12].

The intestinal microcirculation can also be impacted by other factors which are not as easily addressed using imaging techniques. The gut is home to a rich, diverse microbiome which can vary during states of disease. Changes in bacterial populations have the potential to impact the immune response and affect the microcirculation. Amedei and Morbidelli reviewed the effects of various circulating metabolites produced by gut microbiota on endothelial cell function [13]. One study reported interactions between the gut microbiome and the endocannabinoid system, leading to barrier dysfunction and increased permeability, both factors which impact microcirculation [14]. Alteration in the microbiome may also produce an inflammatory response, which can impact the microcirculation. Tissue oxygenation is also an important aspect of intestinal microcirculation and can be considered as an additional functional measure to evaluate microcirculatory changes. As an example, gastric tonometry evaluates levels of carbon dioxide in the intestine as a surrogate indicator for blood flow. Near-infrared spectroscopy also assesses tissue oxygenation, by monitoring changes in oxygen availability. These measures provide additional valuable information, but the focus of this review is on imaging techniques for the evaluation of microcirculatory changes.

Various imaging techniques are used to assess the microcirculation experimentally. Intravital microscopy (IVM) is an experimental technique that allows for the visualization of biological processes in vivo. The term encompasses various types of microscopy which can be used for in vivo visualization. IVM typically images tissue architecture and functions by fluorescently labeling components of interest. Various methodologies used in IVM are reviewed by Weigert et al. [15]. IVM can be used to examine cellular responses over time, allowing for the real-time observation of the activity of a single cell. A major advantage of IVM is that it allows these processes to be studied in vivo, maintaining a near-physiological state. The earliest forms of IVM were able to detect blood flow within individual microvessels and leukocyte extravasation [16,17]. More specifically, IVM can be used to identify changes in levels of rolling and adherent leukocytes involved in the process of extravasation. The process of leukocyte migration is described in detail by Nourshargh and Alon [18]. Recently, IVM has been used to examine the fate of individual cells, including division, migration, communication and death [19]. These processes are particularly relevant to the study of infection, inflammation, and cancer. IVM has the potential to address questions that cannot be directly studied in patients, particularly with respect to intestinal microcirculatory changes.

This review identifies some of the changes within the intestinal microcirculation during both acute and chronic inflammation. Acute pathologies addressed include various acute infections, such as infection with *Helicobacter pylori* and rotavirus, as well as severe acute pancreatitis (SAP), necrotizing enterocolitis (NEC) and sepsis. Inflammatory bowel disease and irritable bowel syndrome are discussed as chronic inflammatory pathologies. The major changes found in each of these conditions are summarized in Table 1 and further discussed in the following sections. Many conditions other than those summarized in this review can impact the microcirculation of the gut. One important example is ischemia-reperfusion injuries, which have recently been reviewed by McDaniel Mims and Groojans [20,21].

## 2. Intestinal Microcirculation during Acute Pathologies

### 2.1. Acute Infections

Infection of the GI tract with an enteric pathogen can result in acute inflammation. Altered microcirculation is a common feature of infection by various pathogens, including bacteria, viruses and parasites [69,70,71]. Evidence suggests screening for early microcirculatory changes may be able to detect an infection in advance of other clinical signs [72]. Early detection may have the ability to influence the course of infection, which is particularly important in vulnerable populations such as neonates [72]. Any infection causing inflammation in the GI tract may have the potential to impact GI microcirculation. *H. pylori* is a common gastrointestinal pathogen, frequently infecting both the stomach and duodenum. As observed mainly by IVM, *H. pylori* infection can impact the microcirculation via changes in blood flow, leukocyte activity and the endothelial cells. Yoshida et al. demonstrated a significant increase in leukocyte adhesion in rat mesenteric venules after superfusion with *H. pylori* [26]. An additional study confirmed these findings and identified significant albumin leakage, which suggested damage to the endothelium [27]. *H. pylori* infection in mice resulted in a 30% reduction in mucosal blood flow as evaluated by laser Doppler [46]. Clinically, *H. pylori* infection was associated with a decrease in mucosal blood flow in patients who had undergone endoscopic mucosal resection [47]. While the effects of *H. pylori* infection discussed here are related to acute infection, it should be noted that this infection often becomes chronic, resulting in the formation of ulcers. 

Infection with rotavirus, a double-stranded RNA virus, serves as an additional example of microcirculatory changes during acute infection. Rotavirus is a common cause of gastroenteritis among children, resulting in vomiting and diarrhea. This infection can impact both the small and large intestine. Intestinal microvascular endothelial cells are able to recognize double-stranded RNA via Toll-like receptor 3, leading to production of proinflammatory interleukin-12 [73]. Infection of neonatal mice, via oral challenge, with rotavirus has been shown to alter villi microcirculation, resulting in early ischemia and villi atrophy. Villi recovery, including hyperemic microcirculation, coincided with recovery from the illness [22]. IVM has further utility in viral infections, as it can be used to visualize host-pathogen interactions in vivo, but this technique is yet to be employed in research on rotavirus infection [74]. 

### 2.2. Severe Acute Pancreatitis

Severe Acute Pancreatitis (SAP) is a serious illness featuring sudden inflammation of the pancreas, often secondary to gallstones or alcohol abuse. SAP has a mortality rate of approximately 40% and often progresses to sepsis and multiorgan failure [75]. The GI tract plays an important role in the pathology of SAP, as intestinal inflammation results in mucosal barrier breakdown and the translocation of bacteria into systemic circulation. Leukocyte infiltration also plays a critical role in SAP pathogenesis. In mice who had SAP induced via taurocholate, leukocyte adhesion and infiltration was shown to mediate tissue injury in the pancreas via IVM and histology samples [76]. Several studies have identified microcirculatory changes within the intestine, though few studies have directly addressed changes in leukocyte adhesion. Mice with cerulein-induced SAP presented increased intestinal permeability, reduced blood flow, hypoxia and overall deterioration of the microvasculature [23]. Intravascular coagulopathy is also common in SAP. The severity of the coagulopathy directly correlates with the level of markers of endothelial dysfunction in adult patients [64]. These changes likely occur early in disease progression. A marked reduction in capillary perfusion can be seen in the mucosal and subserosal colonic layers of rats as early as six hours after induction of mild pancreatitis using intravenous cerulein [48]. Currently, standard therapies for pancreatitis address the underlying condition and include fluid resuscitation and nutritional support. The use of short peptide-based enteral nutrition slows the progression of microcirculatory dysfunction and protects mucosal barrier integrity in mice with SAP [23]. Other targeted therapies have been tested in animal models. In rats with SAP induced by injection of sodium taurocholate into the pancreatic duct, hydrocortisone reduced glycocalyx degradation and improved microvascular perfusion, as evaluated by laser Doppler imaging [44]. Various vasoactive mediator blockades have also been able to ameliorate dysfunction in similar murine models of SAP [28]. Despite these successes, microcirculation-targeted therapies have yet to be successful in clinical trials [77].

### 2.3. Necrotizing Enterocolitis

Necrotizing Enterocolitis (NEC) is an inflammatory condition that primarily affects the intestine of preterm infants. The intestinal microcirculation of infants is particularly vulnerable to disease due to the variety of age-dependent changes that occur during the postnatal period [78]. The defective development of the intestinal microvasculature, as seen in premature infants, may play a key role in the development of NEC [79]. Using experimental IVM, Sprague-Dawley rat pups with asphyxia/stress-induced NEC were seen to have significantly smaller arterioles compared to control animals, which also featured a distinct stop and go pattern of blood flow [49]. Experimental studies suggest that intestinal ischemia may be furthered by secondary vasoconstriction from a decrease in vasodilator nitric oxide production and an increase in production and response to vasoconstrictor endothelin-A [50,51]. Despite the described role of microcirculatory disturbances in NEC, abdominal ultrasound for the detection of bowel segments with absent perfusion has low sensitivity for the disease in patients [80]. NEC patients exhibit additional hematological abnormalities, including disseminated intravascular coagulation and changes in leukocyte counts [65]. Further studies are needed to determine the utility of monitoring changes in the intestinal microcirculation for NEC management.

### 2.4. Sepsis

Sepsis is a complex syndrome, defined as life-threatening organ dysfunction caused by the dysregulated host response to infection [81]. With over 30 million cases and five million deaths annually, sepsis represents a major global health burden [82]. Septic shock is a subset of sepsis which features profound circulatory and cellular abnormalities, and is associated with increased mortality [81]. Despite many advancements in the understanding of the pathophysiology of sepsis, treatment options are still very limited, with no sepsis-specific therapies currently approved.

A systemic inflammatory response occurs early in sepsis. The innate immune system initiates a vigorous immune response, including the release of proinflammatory cytokines and the recruitment of leukocytes to the affected area. This immune response is dysregulated and can lead to tissue damage, edema and ultimately organ failure [83]. Patients who survive the initial hyperinflammatory phase will often have a compensatory anti-inflammatory response [84]. Reduced levels of inflammatory cell markers occurring during this response are associated with a poor prognosis [85]. IVM has proven a useful tool for examining leukocyte activity in sepsis. Lipopolysaccharide (LPS) administration is frequently used to induce endotoxemia and experimentally assess the inflammatory aspects of sepsis without an active infection. Mice with endotoxemia have a thin, hyperpermeable glycocaly, and a significant increase in leukocyte-endothelial interactions [29]. In vitro analysis of interactions between human endothelial cells and activated neutrophils indicates that L-selectin shedding and integrin dysfunction cause leukocytes to favor alternative sites for attachment, which feature an injured endothelium and reduced perfusion [32]. Leukocyte activity can be maladaptive, as it impairs the response to local infections and further damages these tissues. Therapies aimed at reducing leukocyte trafficking may help to preserve organ function in sepsis [86].

The accurate identification of organ dysfunction is a current priority in sepsis research [87]. Sepsis is currently identified using the sequential organ failure assessment (SOFA) or the quick SOFA, which incorporate clinical findings to give an overall score for organ dysfunction [88,89]. This dysfunction is secondary to the profound changes in blood flow and microcirculation present during sepsis. Animal models of sepsis, including cecal ligation and puncture, and colon-ascendens stent peritonitis, have been instrumental in describing these changes in detail. Sepsis is associated with ubiquitous changes in microcirculation, including decreased capillary density, heterogenous flow within visualized capillaries and mismatched local oxygen supply and demand [90,91]. Studies in septic rats suggest these changes may occur early, before the presentation of macrocirculatory dysfunction [52,53]. These findings are mimicked clinically. While these microcirculatory changes rapidly resolve in patients who survive sepsis, they are persistent in those who ultimately succumb to the disease [92]. As reviewed by De Backer et al., these alterations may be due to acute changes in endothelial function, vasoconstriction, glycocalyx or interactions with circulating cells [90]. Hypoperfusion in the intestine may lead to endothelial damage and loss of barrier function, resulting in the translocation of gut bacteria into systemic circulation and further aggravation of the immune response.

Hemodynamic resuscitation, a standard therapy for septic patients, is associated with reduced mortality [93]. Obonyo et al. assessed the impact of resuscitation on endotoxemia models of sepsis, with over half of included studies identifying an improvement in microcirculatory function [94]. Interestingly, one experimental study reported improvements in sublingual and serosal intestinal microcirculation, but persistent dysfunction within the intestinal mucosal villi [54]. A similar dissociation between the sublingual and intestinal microcirculation was also observed clinically in postoperative septic patients [55]. These improvements may also depend on the stage of sepsis. Ergin et al. observed promising results in terms of parameters including oxygenation and acidosis in early sepsis in endotoxemic rats, but fluid resuscitation proved ineffective in later stages [95]. Patients who die of sepsis are frequently immunosuppressed and have changes in cytokine secretion, enhanced expression of inhibitory ligands and expansion of suppressor cell types [95]. These patients often go into shock and experience a serious drop in blood pressure. In later phases of septic shock, microcirculatory dysfunction is more heterogenous and less likely to respond to fluid resuscitation [96].

Other microcirculation-targeted therapies for sepsis have reached the clinical trial stage, with limited success. Inhaled nitric oxide was unable to correct microcirculatory parameters in septic patients despite macrocirculatory stabilization as assessed by sidestream darkfield videomicroscopy of the sublingual microcirculation [97]. Administration of other drugs with hemodynamic actions, such as nitroglycerin, had mixed results [98,99]. Despite some success, including clinical trials with levosimendan and dobutamine, none of these treatments are approved for clinical use [100]. Other novel ideas include the administration of angiopoietin-2 to stabilize the vascular endothelium and decrease capillary leakage [101]. Statins, which have anti-inflammatory activity, have also been suggested to ameliorate endothelial dysfunction and neuroinflammation, as assessed by IVM of the brain of septic mice [102]. Statins have also recently been shown to improve microvascular oxygenation of rats with colon-ascendens stent peritonitis, without changes to microvascular blood flow [24]. Antioxidative therapies have recently been under investigation for their ability to reduce the tissue damage produced by the early hyperactivation seen in sepsis [103]. Iron chelation therapy reduces the iron-catalyzed production of reactive oxygen species (ROS), both decreasing leukocyte activation and improving some microcirculatory parameters, as evaluated by IVM, in murine models of sepsis [30,31].

## 3. Intestinal Microcirculation during Chronic Pathologies

### 3.1. Inflammatory Bowel Disease

Inflammatory Bowel Disease (IBD) is an umbrella term that describes inflammatory disorders affecting the GI tract. There are two major types: Crohn’s disease (CD) and ulcerative colitis (UC), which are distinguished primarily by the portion of the GI tract affected. While CD may impact any part of the GI tract, UC is typically restricted to the large intestine. In addition, CD is a transmural disease, affecting all layers of the gut, while UC is restricted to the mucosal layer. It is important to consider these specifications when considering the impact of these conditions on the intestinal microcirculation. Despite these differences, both diseases feature symptoms including abdominal pain, diarrhea, fatigue and weight loss. One recent global meta-analysis suggests that the prevalence of IBD exceeds 0.3% of the population in Westernized countries [104]. IBD is thought to be caused by the inappropriate mucosal immune response to normal intestinal flora, leading to chronic inflammation and tissue damage. A leaky intestinal epithelium, caused by changes in endothelial function and ion transport, may play an important role in IBD [105].

Different patterns of vascular perfusion have been linked to different stages of IBD progression. Historically these changes were hypothesized to be secondary to histological changes. Animal models, which allow the investigation of very early changes during the disease course, have been essential to this research. In experimental UC induced by either trinitrobenzensulfonic acid or mitomycin-C, perfusion is increased in the early stages of disease and then reduced in chronic phases [56]. These functional changes appear prior to histological damage in these murine UC models [25,56]. Similar findings have been observed in both CD and UC patients, where more mild disease states were associated with increased perfusion and then decreased perfusion in more serious cases [58]. In contrast, Harris et al. found that the reconstitution of recombination-activating gene-deficient mice with T-lymphocytes in a T-cell transfer model of experimental IBD produced two distinct patterns of inflammation: intense inflammation with above normal blood flow, and mild inflammation with below normal blood flow [57]. Changes in perfusion appeared to be dependent on both the level and duration of inflammation experienced.

Chronic inflammation, characteristic of IBD, results in important changes within the intestinal microvasculature which may impact perfusion. Chronic inflammation and hypoxia within the GI tract upregulate various growth factors and other cytokines involved in angiogenesis. Vascular endothelial factor A (VEGF-A) has been linked to both inflammation and angiogenesis in samples from patients with IBD [66]. Increased microvessel density has been demonstrated in IBD patients via both immunohistochemical analysis and imaging methods [59,60]. Because angiogenesis can promote inflammation, therapies directed at this process are of interest. Experimentally, mice deficient in factors associated with angiogenesis, such as cluster of differentiation 40 and its ligand, are resistant to dextran sodium sulfate-induced colitis [68]. Anti-VEGF antibodies have had success in experimental colitis models, reducing both angiogenesis and inflammation [67]. In contrast to this, blockade of the VEGF receptor-3 in interleukin-10 deficient mice with spontaneous colitis impairs lymphatic function and increases inflammation within the colon, but has no significant impact on blood vessels [106]. While anti-VEGF agents are considered safe for use in IBD patients, these agents are not currently used to treat IBD outside of the context of colon cancer [107]. Further, while angiogenesis promotes new blood vessel formation, this increase in volume does not always compensate for other microvascular changes. While there is a volumetric increase in experimental UC, IVM shows an overall reduction in flow velocity which facilitates leukocyte adhesion within microvessels [61]. Human intestinal mucosal microvascular cells, isolated from IBD patients, also exhibit enhanced leukocyte adhesion independent of these changes in flow velocity [33]. In patients with CD, MR imaging showed increased permeability and limited tissue flow despite increased vessel density [45].

Because IBD patients experience chronic inflammation, there is involvement of the adaptive immune system. Lymphocyte homing is of particular relevance. Patients with IBD express high levels of chemokine ligand 20 (CCL20), which facilitates lymphocyte homing to chemokine receptor 6 in intestinal tissues [35,36]. Increased levels of both T and B cell trafficking to the colonic mucosa have been observed via IVM in mice with dextran sodium sulfate-induced colitis, related to the increased expression of CCL20 [37]. Administration of a monoclonal anti-CCL20 antibody, or receptor desensitization, significantly reduced lymphocyte accumulation in these mice [37]. Other adhesion molecules, including mucosal addressin cell adhesion molecule-1 and vascular cell adhesion molecule-1, are also upregulated in the inflamed colon, which promote the recruitment of both lymphocytes and granulocytes [34,38]. This upregulation contributes to the ongoing inflammation within the tissues. The trafficking mechanisms of other leukocytes within the small intestine and colon are reviewed in detail by Habtezion et al. [108]. Blockades of adhesion molecules are not always clinically effective but remain a strategy of interest for the treatment of IBD in patients [109].

Once patients enter remission, it is unclear what happens within the microcirculation. One macroscopic study indicated patients primarily experience a return to normal blood flow, with differences in flow possibly predicting treatment outcomes [110]. A study by Tian et al. noted that an imbalance in microcirculation may still exist in patients in remission, suggesting that blood flow patterns may be used as an indicator of mucosal healing [62]. Patients in remission appear to have normal intestinal blood flow from a macroscopic perspective, as assessed by splanchnic flowmetry, with the absence of normal blood flow serving as a potential indicator of early relapse [111]. Relapses are common, with over half of IBD patients experiencing at least one relapse after the discontinuation of anti-inflammatory therapy infliximab [112]. Most of these patients, though asymptomatic, still had a degree of mucosal inflammation [39]. This is further supported by evidence which suggested that patients in remission had increased levels of aggregated leukocytes within their peripheral blood [40].

### 3.2. Irritable Bowel Syndrome

Irritable bowel syndrome (IBS) is a functional disorder of the GI tract which has a prevalence of approximately 12% in North America [113]. IBS is a chronic, relapsing condition featuring abdominal pain and changes in defecation habits. Unlike IBD, IBS is characterized by a lack of visible abnormalities within the colon and, therefore, is typically considered to be a functional disorder. Various mechanisms of pathogenesis have been posited, including changes in motility, intestinal flora, pain perception or low grade inflammation [114]. Because of the relative lack of understanding of the pathology of IBS, therapeutic options are also lacking.

Visceral hypersensitivity, as a result of autonomic dysfunction, has been suggested as a possible mechanism for IBS [115]. Fingertip blood flow, which can be assessed with laser Doppler perfusion imaging, has been used as an indicator of autonomic function in patients with IBS. Abnormal fingertip blood flow suggests excess sympathetic activity [116]. While these changes in autonomic function have not been linked directly to changes in intestinal microcirculation, several studies have linked autonomic dysfunction to microcirculatory changes in other organs. As an example, patients with familial dysautonomia were observed via laser Doppler imaging to have increased baseline perfusion in the midclavicular area, which may indicate dilation of the microvasculature [117]. In the feet of diabetic patients, early manifestations of autonomic dysfunction are also correlated with impaired vascular tone [118]. In addition, vasoactive intestinal peptide levels are elevated in IBS patients [63]. Administration of vasoactive intestinal peptide has resulted in vasodilation and increased functional capillary density in other experimental models of intestinal microcirculatory dysfunction [119,120]. Based on this evidence, it is reasonable to speculate that autonomic dysfunction may result in altered microcirculatory flow in the intestine of IBS patients.

Chronic, low level inflammation may play a role in IBS pathogenesis [121]. Experimental colonic hyperalgesia, induced by either inflammatory stimuli (zymosan or trinitrobenzensulfonic acid) or high anxiety Wistar-Kyoto rats, can be ameliorated with the inhibition of endothelial cell adhesion molecule expression [41]. This suggests a potential benefit to reducing the number of inflammatory cells migrating into tissues. Additionally, mast cells are increased in both the small and large intestine during IBS [42,43]. Nickel-sensitive patients exhibit IBS-like symptoms including enhanced mucosal perfusion, as assessed by laser Doppler perfusion imaging, which result from increased Toll-like receptor 4 (TLR4) activation and downstream signaling [122]. While nickel sensitivity is a differential diagnosis for IBS, it can give insight into possible changes which occur as a result of inflammation during IBS. TLR4 levels are increased in IBS patients, which suggests some degree of immune dysfunction is occurring [123]. TLR4 activation has also been shown to impair the intestinal microcirculation in experimental models of endotoxemia, acute pancreatitis and NEC [51,124,125]. As assessed with IVM, TLR4 blockade can attenuate changes in functional capillary density in the microcirculation of endotoxemic rats [124]. While the role of inflammation in IBS requires further investigation, current evidence suggests that anti-inflammatory therapies may have some utility. New perspectives in IBS therapies, including the usage of anti-inflammatory corticosteroids, antibiotics, mast cell stabilizers and aminosalicylates, are reviewed by Sinagra et al. [126].

## 4. Conclusions

Microcirculatory dysfunction resulting from inflammatory illnesses is a serious clinical concern associated with negative outcomes including tissue damage and organ dysfunction. Despite the variety of clinical conditions discussed here, inflammatory conditions impacting the intestinal microcirculation have many common characteristics regardless of whether the inflammation is acute or chronic in nature (Table 1). Microcirculatory dysfunction is frequently associated with changes in coagulation, mucosal barrier structure/function and vasoconstriction. Increased leukocyte adhesion has also been identified in many of these conditions. These mechanisms remain similar regardless of the portion of the intestine affected and can be maladaptive and damaging to the patient. As such, many therapies of interest have been trialed in multiple conditions featuring microcirculatory dysfunction. For most of these conditions, these therapies have not been successfully translated from animal models to clinical application. The heterogenous nature of these conditions, and the multitude of factors at play within the microcirculation, complicate this process. IVM remains a useful method for both deepening our understanding of the mechanisms behind microcirculatory dysfunction and the discovery of novel therapies in animals, but care must be taken to help ensure translatability to a human population.

## Figures and Tables

**Table 1:** Possible microcirculatory alterations of the intestine are listed, along with associated conditions, as discussed in this review.

Microcirculatory Alteration	Condition	Type	Methodology	Main Findings	Reference
Ischemia/hypoxia	Rotavirus	Preclinical	Morphometry	Early ischemia in intestinal villi	[22]
	SAP	Preclinical	RT-PCR	Intestinal hypoxia, high expression of hypoxic factors	[23]
	Sepsis	Preclinical	Spectrophotometry	Decreased microcirculatory oxygenation	[24]
	IBD ^1^	Preclinical	Protein expression	Ischemic lamina propria	[25]
Villi atrophy	Rotavirus	Preclinical	Histochemical techniques	Short, ischemic villi early in illness; hyperemic microcirculation on recovery	[22]
Leukocyte activity	*H. pylori*	Preclinical	IVM ^2^	Increased leukocyte adhesion	[26,27]
	SAP ^3^	Preclinical	IVM	Increased leukocyte rolling	[28]
	Sepsis	Preclinical	IVM, in vitro assay	Increased leukocyte and adhesion, maldistribution to damaged endothelium	[29,30,31,32]
	IBD	Clinical	Ex vivo assay	Patient cells exhibit increased leukocyte-binding capacity	[33,34]
		Preclinical	FACS ^4^, flow cytometry	Increased lymphocyte homing	[35,36]
		Preclinical	IVM, immunohistochemistry	Increased lymphocyte adhesion, facilitated by adhesion molecules and chemokines	[37,38]
		Clinical	Endoscopy, histology	Inflammation persists into remission	[39]
		Clinical	Leukocyte adhesion/aggregation test	Increased adhesiveness persists into remission	[40]
	IBS ^5^	Preclinical	Physical response	Inhibition of leukocyte adhesion molecules ameliorated disease	[41]
		Clinical	Cell count, immunohistochemistry	Increased mast cells	[42,43]
Barrier dysfunction	*H. pylori*	Preclinical	IVM	Increased microvascular albumin leakage	[27]
	SAP	Preclinical	Fluorescence, electron microscopy	Increased intestinal permeability; degradation of the glycocalyx	[23,28,44]
	Sepsis	Preclinical	IVM, electron microscopy	Reduced thickness of the glycocalyx, molecular hyperpermeability	[29]
	IBD	Preclinical	Evan’s blue dye	Increased vascular permeability over time	[25]
		Clinical	MR imaging	Vessel permeability increases with disease chronicity	[45]
Capillary perfusion	*H. pylori*	Preclinical	Laser Doppler	30% reduction in mucosal blood flow	[46]
		Clinical	Laser Doppler	Decreased mucosal blood flow	[47]
	Rotavirus	Preclinical	Histochemical techniques	Reduced blood flow in early infection	[22]
	SAP	Preclinical	Laser Doppler, IVM	Impaired mucosal microcirculation; reduced colonic perfusion	[23,28,44,48]
	NEC ^6^	Preclinical	IVM, laser Doppler	“Stop and go” arteriole flow, reduced microvascular perfusion and intestinal flow	[49,50]
		Preclinical	Confocal microscopy	Intestinal perfusion reduced with TLR4 ^7^ activation	[51]
	Sepsis	Preclinical	Side-stream darkfield imaging	Impaired microvascular flow index despite macrovascular parameters; persistent dysfunction in mucosal villi	[52,53,54]
		Preclinical	IVM	Decreased capillary density	[30,31]
		Clinical	Side-stream darkfield imaging	Sublingual perfusion not correlated to intestinal perfusion	[55]
	IBD	Preclinical	IVM	Perfusion varies with disease stage	[56]
		Preclinical	Fluorescent angiography, laser Doppler	Perfusion abnormalities precede histological abnormalities, mild inflammation associated with decreased blood flow	[25,57]
		Clinical	^83^Kr-clearance	Mild disease associated with increased perfusion, severe associated with decreased	[58]
		Clinical	Immunostaining, Narrow band imaging, MR imaging	Increased microvessel density; reduced perfusion despite increased vessel density	[45,59,60]
		Preclinical	IVM, cellular topographic mapping	Reduced velocity despite volumetric increase in flow	[61]
		Clinical	Confocal laser endomicroscopy	UC ^8^ patients in remission had reduced mucosal capillary density	[62]
	IBS	Preclinical	Immunoassay	Increased levels of vasoactive intestinal peptides may impact blood flow	[63]
Microvessel structure	NEC	Preclinical	IVM	Arterioles reduced in size	[49]
	IBD	Preclinical	Histology	Vessel stenosis; dilated vessels in the lamina propria and submucosa	[25]
Hematological abnormalities,	SAP	Clinical	Routine hemostasis tests	Patients had at least one abnormal result, some overt DIC ^9^	[64]
Intravascular coagulopathy	NEC	Clinical	Review	Thrombocytopenia, altered neutrophil counts, hemolytic anemia. DIC	[65]
	IBD	Preclinical	Laser Doppler	Reduced red blood cell concentration	[57]
Angiogenesis	IBD	Clinical	Flow cytometry, immunohistochemistry., cytokine release	Increased expression of angiogenic factors	[59,66,67,68]

^1^ Inflammatory bowel syndrome; ^2^ Intravital microscopy; ^3^ Severe acute pancreatitis; ^4^ Fluorescence-activated cell sorting; ^5^ Irritable bowel syndrome. ^6^ Necrotizing enterocolitis; ^7^ Toll-like receptor 4; ^8^ Ulcerative colitis; ^9^ Diffuse intravascular coagulation.

## Abbreviations

FCD	Functional capillary density
GI	Gastrointestinal
IVM	Intravital microscopy
LPS	Lipopolysaccharide
SAP	Severe acute pancreatitis
NEC	Necrotizing enterocolitis
SOFA	Sequential organ failure assessment
ROS	Reactive oxygen species
IBD	Inflammatory bowel disorder
CD	Crohn’s disease
UC	Ulcerative colitis
VEGF-A	Vascular endothelial growth factor A
CCL20	Chemokine ligand 20
IBS	Irritable bowel syndrome
TLR4	Toll-like receptor 4

## References

[1] Guven G., Hilty M.P., Ince C. (2020). Microcirculation: Physiology, Pathophysiology, and Clinical Application. Blood Purif..

[2] Matheson P.J., Wilson M.A., Garrison R.N. (2000). Regulation of intestinal blood flow. J. Surg. Res..

[3] Zheng L., Kelly C.J., Colgan S.P. (2015). Physiologic hypoxia and oxygen homeostasis in the healthy intestine. A review in the theme: Cellular responses to hypoxia. Am. J. Physiol. Cell Physiol..

[4] Xu J., Ma L., Sun S., Lu X., Wu X., Li Z., Tang W. (2013). Fluid resuscitation guided by sublingual partial pressure of carbon dioxide during hemorrhagic shock in a porcine model. Shock.

[5] Ince C. (2015). Hemodynamic coherence and the rationale for monitoring the microcirculation. Crit. Care.

[6] McDonald D.M. (2008). Angiogenesis and vascular remodeling in inflammation and cancer: Biology and architecture of the vasculature. Angiogenesis: An Integrative Approach from Science to Medicine.

[7] Boerma E.C., van der Voort P.H.J., Spronk P.E., Ince C. (2007). Relationship between sublingual and intestinal microcirculatory perfusion in patients with abdominal sepsis. Crit. Care Med..

[8] Kiessling A.-H., Reyher C., Philipp M., Beiras-Fernandez A., Moritz A. (2015). Real-time measurement of rectal mucosal microcirculation during cardiopulmonary bypass. J. Cardiothorac. Vasc. Anesth..

[9] Do Amaral Tafner P.F., Chen F.K., Filho R.R., Corrêa T.D., De Freitas Chaves R.C., Neto A.S. (2017). Recent advances in bedside microcirculation assessment in critically ill patients. Rev. Bras. Ter. Intensiva.

[10] Ocak I., Kara A., Ince C. (2016). Monitoring microcirculation. Best Pract. Res. Clin. Anaesthesiol..

[11] Ince C., Boerma E.C., Cecconi M., De Backer D., Shapiro N.I., Duranteau J., Pinsky M.R., Artigas A., Teboul J.L., Reiss I.K.M. (2018). Second consensus on the assessment of sublingual microcirculation in critically ill patients: Results from a task force of the European Society of Intensive Care Medicine. Intensive Care Med..

[12] Sharawy N., Mukhtar A., Islam S., Mahrous R., Mohamed H., Ali M., Hakeem A., Hossny O., Refaa A., Saka A. (2017). Preliminary clinical evaluation of automated analysis of the sublingual microcirculation in the assessment of patients with septic shock: Comparison of automated versus semi-automated software. Clin. Hemorheol. Microcirc..

[13] Amedeo A., Morbidelli L. (2019). Circulating Metabolites Originating from Gut. Molecules.

[14] Muccioli G.G., Naslain D., Bäckhed F., Reigstad C.S., Lambert D.M., Delzenne N.M., Cani P.D. (2010). The endocannabinoid system links gut microbiota to adipogenesis. Mol. Syst. Biol..

[15] Weigert R., Sramkova M., Parente L., Amornphimoltham P., Masedunskas A. (2010). Intravital microscopy: A novel tool to study cell biology in living animals. Histochem. Cell Biol..

[16] Hoole S., van Leeuwenhoek A. (2011). The select works of Antony van Leeuwenhoek: Containing his microscopical discoveries in many of the works of nature / translated from the Dutch and Latin editions published by the author, by Samuel Hoole. London G. Sidney.

[17] Dutrochet H. (1826). Recherches Anatomiques et Physiologiques sur la Structure Intime des Animaux et des Vegetaux, et sur Leur Motilite. Edinburgh Med. Surg. J..

[18] Nourshargh S., Alon R. (2014). Leukocyte Migration into Inflamed Tissues. Immunity.

[19] Pittet M.J., Weissleder R. (2011). Intravital imaging. Cell.

[20] McDaniel Mims B., Grisham M.B. (2018). Humanizing the mouse immune system to study splanchnic organ inflammation. J. Physiol..

[21] Grootjans J., Lenaerts K., Buurman W.A., Dejong C.H.C., Derikx J.P.M. (2016). Life and death at the mucosal-luminal interface: New perspectives on human intestinal ischemia-reperfusion. World J. Gastroenterol..

[22] Osborne M.P., Haddon S.J., Worton K.J., Spencer A.J., Starkey W.G., Thornber D., Stephen J. (1991). Rotavirus-induced changes in the microcirculation of intestinal villi of neonatal mice in relation to induction and persistance of diarrhea. J. Pediatr. Gastroenterol. Nutr..

[23] Zhang J., Yu W.Q., Wei T., Zhang C., Wen L., Chen Q., Chen W., Qiu J.Y., Zhang Y., Liang T.B. (2020). Effects of Short-Peptide-Based Enteral Nutrition on the Intestinal Microcirculation and Mucosal Barrier in Mice with Severe Acute Pancreatitis. Mol. Nutr. Food Res..

[24] Schulz J., Vollmer C., Truse R., Bauer I., Beck C., Picker O., Herminghaus A. (2020). Effect of Pravastatin Pretreatment and Hypercapnia on Intestinal Microvascular Oxygenation and Blood Flow during Sepsis. Shock.

[25] Saijo H., Tatsumi N., Arihiro S., Kato T., Okabe M., Tajiri H., Hashimoto H. (2015). Microangiopathy triggers, and inducible nitric oxide synthase exacerbates dextran sulfate sodium-induced colitis. Lab. Investig..

[26] Yoshida N., Granger D.N., Evans D.J., Evans D.G., Graham D.Y., Anderson D.C., Wolf R.E., Kvietys P.R. (1993). Mechanisms involved in Helicobacter pylori-Induced inflammation. Gastroenterology.

[27] Kurose I., Granger D.N., Evans D.J., Evans D.G., Graham D.Y., Miyasaka M., Anderson D.C., Wolf R.E., Cepinskas G., Kvietys P.R. (1994). Helicobacter pylori-induced microvascular protein leakage in rats: Role of neutrophils, mast cells, and platelets. Gastroenterology.

[28] Eibl G., Buhr H.J., Foitzik T. (2002). Therapy of microcirculatory disorders in severe acute pancreatitis: What mediators should we block?. Intensive Care Med..

[29] Kataoka H., Ushiyama A., Akimoto Y., Matsubara S., Kawakami H., Iijima T. (2017). Structural Behavior of the Endothelial Glycocalyx Is Associated with Pathophysiologic Status in Septic Mice: An Integrated Approach to Analyzing the Behavior and Function of the Glycocalyx Using Both Electron and Fluorescence Intravital Microscopy. Anesth. Analg..

[30] Fokam D., Dickson K., Kamali K., Holbein B., Colp P. (2020). Iron Chelation in Murine Models of Systemic Inflammation Induced by Gram-Positive and Gram-Negative Toxins. Antibiotics.

[31] Islam S., Jarosch S., Zhou J., Parquet M.D.C., Toguri J.T., Colp P., Holbein B.E., Lehmann C. (2015). Anti-inflammatory and anti-bacterial effects of iron chelation in experimental sepsis. J. Surg. Res..

[32] Ploppa A., Schmidt V., Hientz A., Reutershan J., Haeberle H.A., Nohé B. (2010). Mechanisms of leukocyte distribution during sepsis: An experimental study on the interdependence of cell activation, shear stress and endothelial injury. Crit. Care.

[33] Binion D.G., West G.A., Ina K., Ziats N.P., Emancipator S.N., Fiocchi C. (1997). Enhanced leukocyte binding by intestinal microvascular endothelial cells in inflammatory bowel disease. Gastroenterology.

[34] Souza H.S., Elia C.C.S., Spencer J., MacDonald T.T. (1999). Expression of lymphocyte-endothelial receptor-ligand pairs, α4β7/MAdCAM-1 and OX40/OX40 ligand in the colon and jejunum of patients with inflammatory bowel disease. Gut.

[35] Kaser A., Ludwiczek O., Holzmann S., Moschen A.R., Weiss G., Enrich B., Graziadei I., Dunzendorfer S., Wiedermann C.J., Mürzl E. (2004). Increased expression of CCL20 in human inflammatory bowel disease. J. Clin. Immunol..

[36] Wang C., Kang S.G., Lee J., Sun Z., Kim C.H. (2009). The roles of CCR6 in migration of Th17 cells and regulation of effector T-cell balance in the gut. Mucosal. Immunol..

[37] Teramoto K., Miura S., Tsuzuki Y., Hokari R., Watanabe C., Inamura T., Ogawa T., Hosoe N., Nagata H., Ishii H. (2005). Increased lymphocyte trafficking to colonic microvessels is dependent on MAdCAM- 1 and C-C chemokine mLARC / CCL20 in DSS-induced mice colitis. Clin. Exp. Immunol..

[38] Nakamura S., Ohtani H., Watanabe Y., Fukushima K., Matsumoto T., Kitano A., Kobayashi K., Nagura H. (1993). In situ expression of the cell adhesion molecules in inflammatory bowel disease: Evidence of immunologic activation of vascular endothelial cells. Lab. Investig..

[39] Baars J.E., Nuij V.J.A.A., Oldenburg B., Kuipers E.J., Van Der Woude C.J. (2012). Majority of patients with inflammatory bowel disease in clinical remission have mucosal inflammation. Inflamm. Bowel Dis..

[40] Arber N., Hallak A., Dotan I., Bujanover Y., Liberman E., Santo M., Moshkowitz M., Tiomny E., Aronson M., Berliner S. (1996). Increased leukocyte adhesiveness/aggregation in patients with inflammatory bowel disease during remission: Further evidence for subclinical inflammation. Dis. Colon Rectum.

[41] Winchester W.J., Johnson A., Hicks G.A., Gebhart G.F., Greenwood-Van Meerveld B., McLean P.G. (2009). Inhibition of endothelial cell adhesion molecule expression improves colonic hyperalgaesia. Neurogastroenterol. Motil..

[42] Robles A., Perez Ingles D., Myneedu K., Deoker A., Sarosiek I., Zuckerman M.J., Schmulson M.J., Bashashati M. (2019). Mast cells are increased in the small intestinal mucosa of patients with irritable bowel syndrome: A systematic review and meta-analysis. Neurogastroenterol. Motil..

[43] O’Sullivan C., Breslin H., O’Morain B.M. (2000). Increased mast cells in the irritable bowel syndrome. Neurogastroenterol. Motil..

[44] Gao S.L., Zhang Y., Zhang S.Y., Liang Z.Y., Yu W.Q., Liang T.B. (2015). The hydrocortisone protection of glycocalyx on the intestinal capillary endothelium during severe acute pancreatitis. Shock.

[45] Taylor S.A., Punwani S., Rodriguez-Justo M., Bainbridge A., Greenhalgh R., De Vita E., Forbes A., Cohen R., Windsor A., Obichere A. (2009). Mural Crohn disease: Correlation of dynamic contrast-enhanced MR imaging findings with angiogenesis and inflammation at histologic examination-Pilot study. Radiology.

[46] Henriksnäs J., Atuma C., Phillipson M., Sandler S., Engstrand L., Holm L. (2009). Acute effects of Helicobacter pylori extracts on gastric mucosal blood flow in the mouse. World J. Gastroenterol..

[47] Adachi K., Suetsugu H., Moriyama N., Kazumori H., Kawamura A., Fujishiro H., Sato H., Okuyama T., Ishihara S., Watanabe M. (2001). Influence of helicobacter pylori infection and cetraxate on gastric mucosal blood flow during healing of endoscopic mucosal resection-induced ulcers. J. Gastroenterol. Hepatol..

[48] Hotz H.G., Foitzik T., Rohweder J., Schulzke J.D., Fromm M., Runkel N.S.F., Buhr H.J. (1998). Intestinal Microcirculation and Gut Permeability in Acute Pancreatitis: Early Changes and Therapeutic Implications. J. Gastrointest. Surg..

[49] Downard C.D., Grant S.N., Matheson P.J., Guillaume A.W., Debski R., Fallat M.E., Garrison R.N. (2011). Altered intestinal microcirculation is the critical event in the development of necrotizing enterocolitis. J. Pediatr. Surg..

[50] Ito Y., Doelle S.M., Clark J.A., Halpern M.D., Mccuskey R.S., Dvorak B. (2007). Intestinal microcirculatory dysfunction during the development of experimental necrotizing enterocolitis. Pediatr. Res..

[51] Yazji I., Sodhi C.P., Lee E.K., Good M., Egan C.E., Afrazi A., Neal M.D., Jia H., Lin J., Ma C. (2013). Endothelial TLR4 activation impairs intestinal microcirculatory perfusion in necrotizing enterocolitis via eNOS-NO-nitrite signaling. Proc. Natl. Acad. Sci. USA.

[52] Dyson A., Cone S., Singer M., Ackland G.L. (2012). Microvascular and macrovascular flow are uncoupled in early polymicrobial sepsis. Br. J. Anaesth.

[53] Hua T., Wu X., Wang W., Li H., Bradley J., Peberdy M.A., Ornato J.P., Tang W. (2018). Micro- and Macrocirculatory Changes During Sepsis and Septic Shock in a Rat Model. Shock.

[54] Dubin A., Edul V.S.K., Pozo M.O., Murias G., Canullán C.M., Martins E.F., Ferrara G., Canales H.S., Laporte M., Estenssoro E. (2008). Persistent villi hypoperfusion explains intramucosal acidosis in sheep endotoxemia. Crit. Care Med..

[55] Edul V.S.K., Ince C., Navarro N., Previgliano L., Risso-Vazquez A., Rubatto P.N., Dubin A. (2014). Dissociation between sublingual and gut microcirculation in the response to a fluid challenge in postoperative patients with abdominal sepsis. Ann. Intensive Care.

[56] Kruschewski M., Foitzik T., Perez-Cantó A., Hũbotter A., Buhr H.J. (2001). Changes of colonic mucosal microcirculation and histology in two colitis models: An experimental study using intravital microscopy and a new histological scoring system. Dig. Dis. Sci..

[57] Harris N.R., Carter P.R., Lee S., Watts M.N., Zhang S., Grisham M.B. (2010). Association between blood flow and inflammatory state in a T-cell transfer model of inflammatory bowel disease in mice. Inflamm. Bowel Dis..

[58] Hultén L., Lindhagen J., Lundgren O., Fasth S., Åhren C. (1977). Regional Intestinal Blood Flow in Ulcerative Colitis and Crohn’s Disease. Gastroenterology.

[59] Alkim C., Savas B., Ensari A., Alkim H., Dagli U., Parlak E., Ulker A., Sahin B. (2009). Expression of p53, VEGF, microvessel density, and cyclin-D1 in noncancerous tissue of inflammatory bowel disease. Dig. Dis. Sci..

[60] Danese S., Fiorino G., Angelucci E., Vetrano S., Pagano N., Rando G., Spinelli A., Malesci A., Repici A. (2010). Narrow-band imaging endoscopy to assess mucosal angiogenesis in inflammatory bowel disease: A pilot study. World J. Gastroenterol..

[61] Ravnic D.J., Konerding M.A., Tsuda A., Huss H.T., Wolloscheck T., Pratt J.P., Mentzer S.J. (2007). Structural adaptations in the murine colon microcirculation associated with hapten-induced inflammation. Gut.

[62] Tian Y., Zheng Y., Teng G., Li J., Wang H. (2019). Imbalanced mucosal microcirculation in the remission stage of ulcerative colitis using probe-based confocal laser endomicroscopy. BMC Gastroenterol..

[63] Del Valle-Pinero A.Y., Sherwin L.A.B., Anderson E.M., Caudle R.M., Henderson W.A. (2015). Altered vasoactive intestinal peptides expression in irritable bowel syndrome patients and rats with trinitrobenzene sulfonic acid-induced colitis. World J. Gastroenterol..

[64] Dumnicka P., Kuśnierz-Cabala B., Sporek M., Mazur-Laskowska M., Gil K., Kuźniewski M., Ceranowicz P., Warzecha Z., Dembiński A., Bonior J. (2017). Serum concentrations of angiopoietin-2 and soluble fms-like tyrosine kinase 1 (SFlt-1) are associated with coagulopathy among patients with acute pancreatitis. Int. J. Mol. Sci..

[65] Song R., Subbarao G.C., Maheshwari A. (2012). Haematological abnormalities in neonatal necrotizing enterocolitis. J. Matern. Neonatal Med..

[66] Scaldaferri F., Vetrano S., Sans M., Arena V., Straface G., Stigliano E., Repici A., Sturm A., Malesci A., Panes J. (2009). VEGF-A Links Angiogenesis and Inflammation in Inflammatory Bowel Disease Pathogenesis. Gastroenterology.

[67] Ardelean D.S., Yin M., Jerkic M., Peter M., Ngan B., Kerbel R.S., Foster F.S., Letarte M. (2014). Anti-VEGF therapy reduces intestinal inflammation in Endoglin heterozygous mice subjected to experimental colitis. Angiogenesis.

[68] Danese S., Scaldaferri F., Vetrano S., Stefanelli T., Graziani C., Repici A., Ricci R., Straface G., Sgambato A., Malesci A. (2007). Critical role of the CD40-CD40-ligand pathway in regulating mucosal inflammation-driven angiogenesis in inflammatory bowel disease. Gut.

[69] Da Silva Watanabe P., Trevizan A.R., Silva-Filho S.E., Góis M.B., Garcia J.L., Cuman R.K.N., Breithaupt-Faloppa A.C., de Mello Gonçales Sant‘Ana D., de Alcantara Nogueira de Melo G. (2018). Immunocompetent host develops mild intestinal inflammation in acute infection with Toxoplasma gondii. PLoS ONE.

[70] Kondo H., Imai Y., Ishikawa T., Tsubota K.I., Yamaguchi T. (2009). Hemodynamic analysis of microcirculation in malaria infection. Ann. Biomed. Eng..

[71] Funaki S., Tokutomi F., Wada-Takahashi S., Yoshino F., Yoshida A., Maehata Y., Miyamoto C., Toyama T., Sato T., Hamada N. (2016). Porphyromonas gingivalis infection modifies oral microcirculation and aortic vascular function in the stroke-prone spontaneously hypertensive rat (SHRSP). Microb. Pathog..

[72] Weidlich K., Kroth J., Nussbaum C., Hiedl S., Bauer A., Christ F., Genzel-Boroviczeny O. (2009). Changes in microcirculation as early markers for infection in preterm infants-an observational prospective study. Pediatr. Res..

[73] Heidemann J., Rüther C., Kebschull M., Domschke W., Brüwer M., Koch S., Kucharzik T., Maaser C. (2007). Expression of IL-12-related molecules in human intestinal microvascular endothelial cells is regulated by TLR3. Am. J. Physiol. Gastrointest. Liver Physiol..

[74] Sewald X. (2018). Visualizing viral infection in vivo by multi-photon intravital microscopy. Viruses.

[75] Popa C.C., Badiu D.C., Rusu O.C., Grigorean V.T., Neagu S.I., Strugaru C.R. (2016). Mortality prognostic factors in acute pancreatitis. J. Med. Life.

[76] Awla D., Abdulla A., Zhang S., Roller J., Menger M.D., Regnér S., Thorlacius H. (2011). Lymphocyte function antigen-1 regulates neutrophil recruitment and tissue damage in acute pancreatitis. Br. J. Pharmacol..

[77] Johnson C.D., Kingsnorth A.N., Imrie C.W., McMahon M.J., Neoptolemos J.P., McKay C., Toh S.K.C., Skaife P., Leeder P.C., Wilson P. (2001). Double blind, randomised, placebo controlled study of a platelet activating factor antagonist, lexipafant, in the treatment and prevention of organ failure in predicted severe acute pancreatitis. Gut.

[78] Nankervis C.A., Reber K.M., Nowicki P.T. (2001). Age-dependent changes in the postnatal intestinal microcirculation. Microcirculation.

[79] Bowker R.M., Yan X., De Plaen I.G. (2018). Intestinal microcirculation and necrotizing enterocolitis: The vascular endothelial growth factor system. Semin. Fetal Neonatal. Med..

[80] Janssen Lok M., Miyake H., Hock A., Daneman A., Pierro A., Offringa M. (2018). Value of abdominal ultrasound in management of necrotizing enterocolitis: A systematic review and meta-analysis. Pediatr. Surg. Int..

[81] Singer M., Deutschman C.S., Seymour C.W., Shankar-Hari M., Annane D., Bauer M., Bellomo R., Bernard G.R., Chiche J.-D., Coopersmith C.M. (2016). The third international consensus definitions for sepsis and septic shock (sepsis-3). J. Am. Med. Assoc..

[82] Fleischmann C., Scherag A., Adhikari N.K.J., Hartog C.S., Tsaganos T., Schlattmann P., Angus D.C., Reinhart K. (2016). Assessment of global incidence and mortality of hospital-treated sepsis current estimates and limitations. Am. J. Respir. Crit. Care Med..

[83] Harrison C. (2010). Sepsis: Calming the cytokine storm. Nat. Rev. Drug Discov..

[84] Bone R.C., Grodzin C.J., Balk R.A. (1997). Sepsis: A new hypothesis for pathogenesis of the disease process. Chest.

[85] Muller Kobold A.C., Tulleken J.E., Zijlstra J.G., Sluiter W., Hermans J., Kallenberg C.G.M., Cohen Tervaert J.W. (2000). Leukocyte activation in sepsis: Correlations with disease state and mortality. Intensive Care Med..

[86] Huynh T., Nguyen N., Keller S., Moore C., Shin M.C., McKillop I.H. (2010). Reducing leukocyte trafficking preserves hepatic function after sepsis. J. Trauma-Inj. Infect. Crit. Care.

[87] Coopersmith C.M., de Backer D., Deutschman C.S., Ferrer R., Lat I., Machado F.R., Martin G.S., Martin-Loeches I., Nunnally M.E., Antonelli M. (2018). Surviving sepsis campaign: Research priorities for sepsis and septic shock. Intensive Care Med..

[88] Vincent J.L., Moreno R., Takala J., Willatts S., De Mendonca A., Bruining H., Reinhart C.K., Suter P.M., Thijs L.G. (1996). The SOFA (Sepsis-related Organ Failure Assessment) score to describe organ dysfunction/failure. On behalf of the Working Group on Sepsis-Related Problems of the European Society of Intensive Care Medicine. Intensive Care Med..

[89] Seymour C.W., Liu V.X., Iwashyna T.J., Brunkhorst F.M., Rea T.D., Scherag A., Rubenfeld G., Kahn J.M., Shankar-Hari M., Singer M. (2016). Assessment of clinical criteria for sepsis for the third international consensus definitions for sepsis and septic shock (sepsis-3). JAMA-J. Am. Med. Assoc..

[90] De Backer D., Donadello K., Taccone F.S., Ospina-Tascon G., Salgado D., Vincent J.-L. (2011). Microcirculatory alterations: Potential mechanisms and implications for therapy. Ann. Intensive Care.

[91] Ellis C.G., Bateman R.M., Sharpe M.D., Sibbald W.J., Gill R. (2002). Effect of a maldistribution of microvascular blood flow on capillary O2 extraction in sepsis. Am. J. Physiol. Hear. Circ. Physiol..

[92] De Backer D., Creteur J., Sakr Y., Vincent J.-L., Dubois M.-J. (2004). Persistent microcirculatory alterations are associated with organ failure and death in patients with septic shock. Crit. Care Med..

[93] Rochwerg B., Alhazzani W., Sindi A., Heels-Ansdell D., Thabane L., Fox-Robichaud A., Mbuagbaw L., Szczeklik W., Alshamsi F., Altayyar S. (2014). Fluid resuscitation in sepsis: A systematic review and network meta-analysis. Ann. Intern. Med..

[94] Obonyo N.G., Fanning J.P., Ng A.S.Y., Pimenta L.P., Shekar K., Platts D.G., Maitland K., Fraser J.F. (2016). Effects of volume resuscitation on the microcirculation in animal models of lipopolysaccharide sepsis: A systematic review. Intensive Care Med. Exp..

[95] Ergin B., Zafrani L., Kandil A., Baasner S., Lupp C., Demirci C., Westphal M., Ince C. (2016). Fully Balanced Fluids do not Improve Microvascular Oxygenation, Acidosis and Renal Function in a Rat Model of Endotoxemia. Shock.

[96] Hernández G., Teboul J.L. (2016). Is the macrocirculation really dissociated from the microcirculation in septic shock?. Intensive Care Med..

[97] Trzeciak S., Glaspey L.J., Dellinger R.P., Durflinger P., Anderson K., Dezfulian C., Roberts B.W., Chansky M.E., Parrillo J.E., Hollenberg S.M. (2014). Randomized Controlled Trial of Inhaled Nitric Oxide for the Treatment of Microcirculatory Dysfunction in Patients With Sepsis. Crit. Care Med..

[98] Boerma E.C., Koopmans M., Konijn A., Kaiferova K., Bakker A.J., Van Roon E.N., Buter H., Bruins N., Egbers P.H., Gerritsen R.T. (2010). Effects of nitroglycerin on sublingual microcirculatory blood flow in patients with severe sepsis/septic shock after a strict resuscitation protocol: A double-blind randomized placebo controlled trial. Crit. Care Med..

[99] Boerma E.C., Ince C. (2010). The role of vasoactive agents in the resuscitation of microvascular perfusion and tissue oxygenation in critically ill patients. Intensive Care Med..

[100] Enrico C., Kanoore Edul V.S., Vazquez A.R., Pein M.C., Pérez de la Hoz R.A., Ince C., Dubin A. (2012). Systemic and microcirculatory effects of dobutamine in patients with septic shock. J. Crit. Care.

[101] Spicer A., Calfee C.S. (2012). Fixing the leak: Targeting the vascular endothelium in sepsis. Crit. Care.

[102] Reis P.A., Alexandre P.C.B., D’Avila J.C., Siqueira L.D., Antunes B., Estato V., Tibiriça E.V., Verdonk F., Sharshar T., Chrétien F. (2017). Statins prevent cognitive impairment after sepsis by reverting neuroinflammation, and microcirculatory/endothelial dysfunction. Brain. Behav. Immun..

[103] Prauchner C.A. (2017). Oxidative stress in sepsis: Pathophysiological implications justifying antioxidant co-therapy. Burns.

[104] Ng S.C., Shi H.Y., Hamidi N., Underwood F.E., Tang W., Benchimol E.I., Panaccione R., Ghosh S., Wu J.C.Y., Chan F.K.L. (2017). Worldwide incidence and prevalence of inflammatory bowel disease in the 21st century: A systematic review of population-based studies. Lancet.

[105] Ricanek P., Lunde L.K., Frye S.A., Støen M., Nygård S., Morth J.P., Rydning A., Vatn M.H., Amiry-Moghaddam M., Tønjum T. (2015). Reduced expression of aquaporins in human intestinal mucosa in early stage inflammatory bowel disease. Clin. Exp. Gastroenterol..

[106] Jurisic G., Sundberg J.P., Detmar M. (2013). Blockade of VEGF receptor-3 aggravates inflammatory bowel disease and lymphatic vessel enlargement. Inflamm. Bowel Dis..

[107] Herrera Gomez R.G., Gallois C., Adler M., Malka D., Planchard D., Pistilli B., Ducreux M., Mir O. (2019). Safety of bevacizumab in cancer patients with inflammatory bowel disease. J. Clin. Oncol..

[108] Habtezion A., Nguyen L.P., Hadeiba H., Butcher E.C. (2016). Leukocyte Trafficking to the Small Intestine and Colon. Gastroenterology.

[109] Cesarini M., Fiorino G. (2013). Leukocyte traffic control: A novel therapeutic strategy for inflammatory bowel disease—An update. Expert Rev. Clin. Immunol..

[110] Saevik F., Nylund K., Hausken T., Odegaard S., Gilja O.H. (2014). Bowel perfusion measured with dynamic contrast-enhanced ultrasound predicts treatment outcome in patients with crohn’s disease. Inflamm. Bowel Dis..

[111] Ludwig D., Wiener S., Brüning A., Schwarting K., Jantschek G., Stange E.F. (1999). Mesenteric blood flow is related to disease activity and risk of relapse in Crohn’s disease: A prospective follow-up study. Am. J. Gastroenterol..

[112] Bots S.J., Kuin S., Ponsioen C.Y., Gecse K.B., Duijvestein M., D’Haens G.R., Löwenberg M. (2019). Relapse rates and predictors for relapse in a real-life cohort of IBD patients after discontinuation of anti-TNF therapy. Scand. J. Gastroenterol..

[113] Lovell R.M., Ford A.C. (2012). Global Prevalence of and Risk Factors for Irritable Bowel Syndrome: A Meta-analysis. Clin. Gastroenterol. Hepatol..

[114] Malagelada J.R., Malagelada C. (2016). Mechanism-Oriented Therapy of Irritable Bowel Syndrome. Adv. Ther..

[115] Manabe N., Tanaka T., Hata J., Kusunoki H., Haruma K. (2009). Pathophysiology underlying irritable bowel syndrome—From the viewpoint of dysfunction of autonomic nervous system activity-. J. Smooth Muscle Res..

[116] Tanaka T., Manabe N., Hata J., Kusunoki H., Ishii M., Sato M., Kamada T., Shiotani A., Haruma K. (2008). Characterization of autonomic dysfunction in patients with irritable bowel syndrome using fingertip blood flow. Neurogastroenterol. Motil..

[117] Weiser M., Hilz M.J., Bronfin L., Axelrod F.B. (1998). Assessing microcirculation in familial dysautonomia by laser Doppler flowmeter. Clin. Auton. Res..

[118] Sun P.C., Chen C.S., Kuo C.D., Lin H.D., Chan R.C., Kao M.J., Wei S.H. (2012). Impaired microvascular flow motion in subclinical diabetic feet with sudomotor dysfunction. Microvasc. Res..

[119] Leister I., Sydow J., Stojanovic T., Füzesi L., Sattler B., Heuser M., Becker H., Markus P.M. (2005). Impact of vasoactive intestinal polypeptide and gastrin-releasing peptide on small bowel microcirculation and mucosal injury after hepatic ischemia/reperfusion in rats. Int. J. Colorectal Dis..

[120] Suzuki H., Noda Y., Paul S., Gao X.-P., Rubinstein I. (1995). Encapsulation of vasoactive intestinal peptide into liposomes: Effects on vasodilation in vivo. Life Sci..

[121] Ng Q.X., Soh A.Y.S., Loke W., Lim D.Y., Yeo W.S. (2018). The role of inflammation in irritable bowel syndrome (IBS). J. Inflamm. Res..

[122] Borghini R., Puzzono M., Rosato E., Di Tola M., Marino M., Greco F., Picarelli A. (2016). Nickel-Related Intestinal Mucositis in IBS-Like Patients: Laser Doppler Perfusion Imaging and Oral Mucosa Patch Test in Use. Biol. Trace Elem. Res..

[123] Koçak E., Akbal E., Köklü S., Ergül B., Can M. (2016). The colonic tissue levels of TLR2, TLR4 and nitric oxide in patients with irritable bowel syndrome. Intern. Med..

[124] Zhou J., Soltow M., Zimmermann K., Pavlovic D., Johnston B., Lehmann C. (2015). Experimental TLR4 inhibition improves intestinal microcirculation in endotoxemic rats. Microvasc. Res..

[125] Li Y., Zhou Z.G., Zhang J., Chen Y.D., Li H.G., Gao H.K., Wang R., Hu T.Z. (2006). Microcirculatory detection of Toll-like receptor 4 in rat pancreas and intestine. Clin. Hemorheol. Microcirc..

[126] Sinagra E., Morreale G.C., Mohammadian G., Fusco G., Guarnotta V., Tomasello G., Cappello F., Rossi F., Amvrosiadis G., Raimondo D. (2017). New therapeutic perspectives in irritable bowel syndrome: Targeting low-grade inflammation, immuno-neuroendocrine axis, motility, secretion and beyond. World J. Gastroenterol..

